# Implication des maris dans les soins anténatals: enquête sur les facilitateurs et les barrières: Cas des habitants de Bosomtwe au Ghana

**DOI:** 10.1186/s12978-022-01506-7

**Published:** 2022-12-01

**Authors:** Anthony Kwame Morgan, Beatrice Aberinpoka Awafo, Theophilus Quartey, Justin Cobbold

**Affiliations:** 1 Department of Geography and Rural Development, Kwame Nkrumah University of Science and Technology, Kumasi, Ghana; 2 Department of Planning, Kwame Nkrumah University of Science and Technology, Kumasi, Ghana

**Keywords:** Antenatal care, Barriers, Facilitators, Male involvement, Pregnancy, Ghana, Soins prénatals/anténatals, Barrières, Facilitateurs, Implication des hommes, Grossesse, Ghana

## Abstract

**Contexte:**

Cet article a abordé les facilitateurs et les obstacles relatifs à l’implication des maris dans les soins prénatals dans le district de Bosomtwe au Ghana, du point de vue des maris, des femmes enceintes avec et sans expérience d'accouchement, des mères allaitantes, des sage-femmes et des accoucheuses traditionnelles.

**Méthodologie:**

L’étude s’est basée sur une conception de recherche qualitative pour recueillir et analyser les données sur les facilitateurs et les obstacles relatifs à l’implication des maris dans les soins prénatals. Le champ d’analyse était constitué de 36 participants, dont 14 maris, 8 femmes enceintes ayant déjà accouché et mères allaitantes, 6 femmes enceintes n’ayant jamais accouché, 6 sage-femmes et 2 accoucheuses traditionnelles—sélectionnés délibérément. Les données de l’étude ont été recueillies par le biais d'entretiens approfondis et analysées par l’approche du contexte.

**Résultats:**

Nombreux facteurs empêchent la participation active des maris aux soins prénatals. Ces facteurs sont d’ordre économique (contraintes de temps), culturels (l’association de la maternité et ses obligations aux femmes) et le système de santé (manque de Services prénatals destinés aux maris et le comportement des personnels de santé). Malgré cela, certains maris ont participé aux soins prénatals en raison de l'importance qu'ils accordent à la santé et à la sécurité de leurs femmes et du fœtus, de l'évolution des rôles de chacun des sexes et des traitements préférentiels reçus par leurs femmes dans les maternités (en raison de la participation de leurs maris aux soins prénatals).

**Conclusion:**

La mise en œuvre des stratégies alternatives, telles que le conseil aux couples, la prolongation des heures d’ouverture des centres de santé pour accueillir les hommes fonctionnaires, est recommandée afin d'offrir aux maris un moyen plus souple et plus attrayant de soutenir leurs femmes pendant la grossesse. Ces efforts doivent être renforcés par tous dans la société en modifiant l’opinion erronée selon laquelle la conception de la grossesse et l’éducation de l’enfant sont les devoirs de la femme seule.

## Abstract (en)

**Background:**

This paper explored the facilitators and barriers to husbands’ involvement in antenatal-related care in the Bosomtwe District of Ghana from the perspectives of husbands, pregnant women with and without delivery experience, nursing mothers, midwives and traditional birth attendants.

**Methods:**

The study relied on the qualitative research design to collect and analyse data on the facilitators and barriers to husbands’ involvement in antenatal-related care. The unit of analysis was made up of 36 participants—husbands (14), pregnant women with delivery experience and, nursing mothers (8), pregnant women without delivery experience (6), male and female midwives (6) and traditional birth attendants (2) who were purposively selected. The study’s data was gathered using in-depth interviews and analysed through the content approach.

**Results:**

Various economic [work and time constraint], cultural [the association of childbearing and its allied duties to women] and health-system factors [lack of antenatal services targeted at husbands and health professionals’ attitude] hinder husbands’ active participation in antenatal care. Despite these, some husbands participated in antenatal care owing to the importance they accord to the health and safety of their wives and the foetus; changing gender roles and preferential treatments received by their wives at antenatal clinics [as a result of the involvement of their husbands in prenatal care].

**Conclusion:**

The implementation of alternative strategies, like, couple counselling, prolonging operating times of health centres to accommodate working men are recommended to provide a more accommodative and attractive avenue for husbands to support their wives during pregnancy. These efforts must be reinforced by the entire society through modifying the “ill-held view” that pregnancy and childcare is the sole duty of a woman.

## Plain language summary

The involvement of males [husbands] in antenatal-related care is noted to have significant impacts on the wellbeing of pregnant women and lessen burden of pregnancy, delivery and childcare. In this study, we examined the opinions of some stakeholders in the space of pregnancy and childcare [husbands, pregnant women with delivery experience, nursing mothers, midwives and traditional birth attendants], regarding the involvement of husbands in antenatal-related care. The study was conducted in the Bosomtwe District of Ghana, West Africa.

The participants were asked to narrate experiences of husbands’ involvement in pregnancy-related care, in addition to the barriers and facilitators of such engagement processes. A total of 36 participants were involved in the study. The narrative approach was used to report the findings.

It was found that male’s involvement in antenatal-related care is minimal in the study area. Factors such as limited time due to economic activities and the responsibility of providing financial resources for the wellbeing of the family and cultural factors in the form of childbearing being a responsibility of women deterred husbands from antenatal-related care. Again, absence of services that target males also served as a barrier. That notwithstanding, the importance attached by husbands to the health and safety of their wives and the foetus, the changing gender roles and preferential treatments given to pregnant women who are accompanied by their husbands facilitated males involvement.

In conclusion, the study has implications for male-friendly antenatal care services development and continuous effort to undo the “cultural-ills” of male’s involvement in pregnancy care in Ghana.

## Background

Until recently, pregnancy and childbirth have been largely viewed as the sphere of women, with men, often relegated to the peripheries [42]. The role of men (husbands) was in the past, limited to providing financial resources and other materials needed and the naming of the baby [6, 9, 25]. The mother takes charge of childcare alongside performing domestic chores with the support of external family members [6, 25, 41, 50]. Despite a gradual dissociation from this norm, this practice persists in some rural communities [25]. However, due to urbanization and the evolution of the nuclear family structure [5, 6, 29], the external family support for household chores and maternal healthcare services is fading away [29]. In this framework, paternal support in pregnancy and maternal care is becoming more relevant in other aspects as against the mere provision of material and financial resources. This has birthed empirical investigations into male's support during pregnancy.

Research into male participation in antenatal-related care [a form of preventive healthcare that entails identifying high-risk pregnancies and educating women in order for them to have a healthy delivery and outcome] has shown that joint decision-making and partnership in pregnancy and maternal healthcare are preferable to an autocratic or a one-sided approach [11, 37, 52]. In Guatemala for instance, men and women alike perceive joint maternal healthcare decision-making as a means of expressing mutual love [12]. Joint decision-making and male’s involvement in pregancy and maternal healthcare was perceived as a desirable approach to sharing blames and celebrating success in Nepal [37]. Nonetheless, Mullany et al. [37] contended that such joint decision-making arrangements turn to be male-dominated. In Nigeria, Falade-Fatila and Adebayo [21] found a high level of husband’s knowledge on antenatal-related care and high support for their partners, with the quest to protect their partners the primary facilitator of their involvement in antenatal-related care [39].

Within the sphere of reproductive health research in Ghana, attention has been focused on males’ involvement in family planning and childcare [1, 18, 28, 29], and less of antenatal-related care. Oppong [41] contends that male support for maternal healthcare in Ghana is low, making women simultaneously care for their children and participate in the labour market. In a study undertaken by Saah et al. [47], they found low male involvement in skilled birth care in the North Dayi District of Ghana while Quarcoo and Tarkang [45] discovered that married men with higher education are more likely to support their partner’s in maternal healthcare. In general, no known study in Ghana has answered the question on "what are the facilitators and barriers to male's involvement in antenatal-related care among married men, and among rural dwellers in particular?" The dearth of literature on the facilitators and barriers to male’s involvement in antenatal-related care among married men in Ghana and for that matter the Bosomtwe District therefore underscored the need for this study. The study is premised on the need to find ways to reduce maternal and neonatal death as enshrined in the Sustainable Development Goals [SDGs]; since, in one accord, countries around the globe are energized and geared towards the attainment of a better world, devoid of inequality of any form [35]. This is heavily linked to the post-2015 development agenda where goal 3 of the 17 development goals focuses on ensuring healthy lives and promoting the well-being for all at all ages. It is believed that the findings from the study will form the basis for future research into the subject matter while providing a framework for public health discourse and policy on promoting the health and wellbeing of pregnant women towards the attainment of targets 3.1 on maternal mortality [reduce worldwide maternal mortality to fewer than 70 per 100,000 live births by 2030] and 3.2 on child and neonatal mortality [end avoidable deaths among newborns and children under the age of five by 2030, with all nations aiming to decrease neonatal mortality to at least 12 per 1000 live births and under-5 mortality to at least 25 per 1000 live births] of the SDGs. Owing to the immense benefits of males’ involvement in antenatal-related care [which includes better health and wellbeing of pregnant women and their foetus in addition to having a better delivery experience and post-delivery wellbeing] [11, 37, 52], policy interventions that emanate from well grounded empirical studies like these are in high demand in Ghana. This study therefore serves as a piecemeal within the various efforts directed at promoting better health outcomes for pregnant women and their foetus. We are of the view that the resultant discussion and recommendations would be absorbed by the appropriate institution for appropriate action.

### Profile of the study area

The Bosomtwe District is one of the thirty (43) Districts in the Ashanti Region, and one of the 260 Metropolitan, Municipal and District Assemblies [MMDAs] in Ghana. The population of the District as of 2010 was 93,910 with 44,793 males (47.7%) and 49,117 females (52.3%). The District is predominantly rural with 69.8% living in rural areas and about 30.2% in the urban areas [22]. The rural nature of the District underscores its heavy dependence on agricultural activities with an estimated employment rate (directly or indirectly) of 62.9% of the active labour force. The health delivery system in the District is made up of sixteen public and private health institutions [six Hospitals, three Health Centres, six Clinics, two Maternity Homes, eight Community Health Planning Services (CHPS) Compounds and a Midwifery Training Institution]. These health facilities are however inadequate to serve the District effectively due to the growing population amidst increasing demand for healthcare services.

## Methods

### Study design

The study employed an exploratory qualitative research design to investigate the barriers and facilitators to husbands’s support for their wives during the uptake of antenatal services in the Bosomtwe District of Ghana from the perspectives of husbands, pregnant women with and without delivery experience, nursing mothers, midwives and traditional birth attendants.

The exploratory qualitative research approach is applicable in studies with inquiry and descriptive objectives and also appropriate in unearthing unfamiliar or insufficiently researched subject matters [17, 24]. The dearth of literature on husbands’ involvement in antenatal-related care in Ghana, particularly with regards to the barriers and facilitators necessitated this study. The authors believe that the exploratory qualitative approach provides a broader understanding of the subject matter, compared to other qualitative study designs. As a consequence, the researchers and the participants were interdependent and mutually engaged throughout the study, and were opened to new knowledge.

### Study participants

The study’s participants were a purposive sample of thirty (36) ‘stakeholders’ with interest and responsibilities toward the support offered by husbands to their wives during antenatal-related care. In qualitative studies, smaller samples are recommended [31] and as such the decision to recruit thirty-six participants. The unit of analysis included husbands (14), pregnant women with delivery experience and nursing mothers (8), pregnant women without delivery experience (6), male and female midwives (6) and traditional birth attendants (TBA) (2). The units of analysis were considered appropriate due to their direct contact and experiences with husband’s support of their wives during antenatal-related care, thus placing them in a position to provide insightful information to that effect. In instances where couples were included, the husband and the wife were separately interviewed to eliminate the potential of the husband influencing the responses of their wife and vice versa. All the participants were between the ages of twenty-five (25) and fifty (55) years.

### Recruitment

The purposive sampling approach was employed in the selection of the unit of analysis. The choice of the sampling technique was premised on the qualitative research design adopted [19, 20] and the need to obtain rich data from persons with much knowledge on the subject matter. With the study's objective established as  indepthly exploring husband's involvement in antenatal-related care, the collection of rich data was of utmost importance. By employing the purposive sampling technique, only persons deemed to be relevant to the study based on their potential to provide worthy information on the subject matter were selected [19].

Of the six hospitals in the district, three were selected. Kuntanase Government Hospital, ST Michael’s Hospital and Divine Mercy Hospital were selected by means of purposive technique. The purposive approach was defined based on three criteria. The Kuntanase Government Hospital was selected due to the need to include a public health facility which is often patronized by most Ghanaians, partly due to its affordability [4, 7, 51]. ST Michael’s Hospital and Divine Mercy Hospital were selected since they represent two different but important streams in the healthcare delivery framework of Ghana. ST Michael’s Hospital and Divine Mercy Hospital hospitals represents a mission health facility and private health facility respectively, meeting the needs of persons who do not want to put up with the long waiting times at public health facilities due to pressure on these facilities [7, 51].

The heterogeneous purposive sampling strategy was used to recruit the study participants. To determine husbands who were fathers or expectant fathers, the potential participants were asked the following question: “Are you married?” This yielded a “yes” or “no” response. For those who answered “yes”, they were further asked, “Are you a father?” “Or are you an expectant father?” These questions also yielded a “yes” or “no” response. Husbands who are fathers or are expectant fathers were selected and included in the sample. Husbands with children or the last child of not more than 6 months at the time of the data collection were included, while those with a child or the last child more than 6 months old were exluded. To reduce the inclinations of recall bias, which is most pronounced when there is a considerable time lag between an incident and the period of recollection [2, 8], husbands with children who were older than 6 months were excluded. To expand the nature of the sampling procedure, husbands were selected from both health facilities and outside the health facilities. Of the 14 husbands recruited in the study, 3 were selected from the health facilities [2 from the Kuntunase Government Hospital, and 1 from ST Michael’s Hospital], while the remaining 9 were recruited from their homes. With the help of community focal persons [assembly members and other respected individuals], a recommendation and list of houses with potential husbands’ matching the study’s inclusion criteria were provided to the team. Based on this information, the team visited these houses and further evaluated their appropriateness, vis–à-vis the inclusion criteria.

Additionally, age [persons aged between 18 and 55 years], level of education [those with no formal education, primary education, junior high school education, high school education, vocational and technical education and those with tertiary education], and nature of employment of the husbands [formal sector or informal sector employment, employed, self-employed and unemployed] were used as criteria to open up the diversity and heterogeneity of the recruited participants. The pregnant women with and without delivery experience, nursing mothers, and the midwives were all selected from the three health facilities. For nursing the mothers, the inclusion criteria were that: (i) the person was married; and (ii) had delivered not more than six months [half a year]. The exclusion of unmarried nursing mothers was premised on their inability to comment on the support provided by their partner—especially when they are not together, while the exclusion of nursing mothers with children more than six months old is to limit the tendencies of recall bias, which is most prevalent when there is a significant time lag between an event and the time of recall [2, 8]. The distribution of the participants within the health facilities are in this manner: Kuntanase Government Hospital [pregnant women with delivery experience and nursing mothers (4), pregnant women without delivery experience (2) and midwives (3)]; ST Michael’s Hospital [pregnant women with delivery experience and nursing mothers (2), pregnant women without delivery experience (2) and midwives (2)]; and Divine Mercy Hospital [pregnant women with delivery experience and nursing mothers (2), pregnant women without delivery experience (2) and midwives (1)]. Table 1 provides a detailed description of the distribution.

**Table 1:** Distribution of participants within the selected Health Facilities

Participants	Name of Health Facility		
	Kuntunase Government Hospital	ST Michael’s Hospital	Divine Mercy Hospital
Pregnant women with delivery experience and nursing mothers	4	2	2
Pregnant women without delivery experience	2	2	2
Midwives	3	2	1

For the pregnant women without delivery experience, inclusion criteria were that she should be married and not less than 12 weeks pregnant [3 months]. The traditional birth attendants in the study were identified following a recommendation by a midwife at the Kuntanase Government Hospital. Two out of the five traditional birth attendants that were recommended were selected based on the longevity of their practice. In all, persons who met the various inclusion criteria among the several categories of the participants were approached and their written consent were sought for participation in the study.

### Data collection

Thirty-six in-depth interviews were conducted between the periods of September and November 2019 and between March 9, 2022 and March 18, 2022. Upon evidence from the literature, a semi-structured interview guide was used to gather the data. This offered the participants the opportunity to express their opinions behaviours and beliefs about the subject matter; a phenomenon that resonates with the qualitative research design [48]. The interview guide were developed in English and translated into Twi (the local language of the study participants). The guide was field-tested with three individuals from the research location who were not part of the participants included in the final analysis. Overall, the field testing helped the researchers to make changes that were required, particularly in the guide structure, sequence, and concepts.

The questions primarily covered facilitators and barriers to male’s involvement in antenatal-related care. For instance, some important questions included in the interview guide for husbands were: “Have you ever supported your wife during pregnancy?” “In What ways do you support your wife during pregnancy? “Why will you accompany your wife to antenatal care?” “Why do husbands find it difficult to support their wives during antenatal care?” “What must be done to encourage the support of husbands toward their wives during pregnancy?” The interviews for the midwives were conducted at the health facilities while that of the husbands, pregnant women with or without delivery experiences, nursing mother and TBAs, were conducted at their respective homes, upon their requests. The interviews were conducted by first and second authors with the help of three other research assistants with extensive expertise in qualitative data collection, are native to the study location, speaks the local language and are well-versed in the local culture of the people. Some interviews were conducted in English language, while others were conducted in Twi. Each interview session lasted for periods between 40 and 60 min. The interviews were audio-recorded and transcribed verbatim. Ultimately the researchers summarized the transcribed interviews to authenticate the content as a procedure of member checking [15]. The interview method was selected for this qualitative study as it provided insight into the perceptions and behaviours of participants, which are embedded in the social and cultural contexts [10, 46].

### Analysis

The content analysis was employed in analysing the transcribed interviews [23]. The transcribed interviews were repetitively read to comprehend the totality of the contents and issues discussed. Subject matters were identified in each transcribed interview and abridged. The condensing units or subject matters were coded and classified. Coding of the data was done by the first and second authors. The codes and classifications were discussed among the authors and with three other persons, with no direct interest in the research, to ensure validity [15]. These other people were people with in-depth knowledge and experience in qualitative research. The purpose of discussing the codes with these experts was to gain additional insight from persons with no interest and persons who could easily identify researcher bias, which is often common in qualitative studies. Their inputs helped in reclassifying some categories that were initially merged. The results were presented under the identified units or subject matters with direct quotations from the participants to support the discussion.

### Ethical consideration

The study adhered to all ethical guidelines. For starters, during the data collecting procedure, the concept of voluntary permission [13] as it relates to social science research was followed in the study. Written informed consents were obtained from the participants before their participation in the data collection. The goal of the research and how the data gathered is to be used were explained to the participants. Second, while publishing the views of the participants, anonymity and privacy were protected.

## Results

The results from the content analysis are presented in the subsequent sections. Specifically, the analysis and the presentation of the results were organized under the following themes; socio-demographic characteristics of the participants, the experiences of male involvement in antenatal-related care among married men in Bosomtwe, facilitators and barriers to male’s involvement in antenatal-related care among married men in Bosomtwe.

### Socio-demographic traits of the participants

The socio-economic and demographic characteristics of the participants revealed that 16 of the participants were males while 20 were females. Within the age distribution, 5 of the participants were within the age ranges of 25–30, and 13 were aged between 31 and 35. Furthermore, 7 were within the age ranges of 36–40 and 8 of them were between the age ranges of 41 and 45 years. The final age bracket, 46-50 had 3 participants. We found that 6 of the participants had no formal education, 8 had basic (primary education), 12 had secondary education, and 10 had tertiary education. With regards to sector of employment, 10 of the participants are public sector employees, 12 of them are employed in the private sector and 14 or them are self-employed. Less than ₵500 (< $67)—2 participants, between ₵500–1000 ($67–$133.3)—5 participants, ₵1001–1500 ($133.4–$200)—9 participants, between ₵1501–2000 ($200.1–$266.7)—14 participants and above ₵2000 (Above $266.7)—6 participants, were the self-reported average monthly income distribution of the participants. Table 2 presents a detailed description of the demographic and socio-economic characteristics of the participants.

**Table 2:** Socio-demographic characteristics of the participants

Variable	Response	Count
Gender	Male	16
	Female	20
	Total	36
Age *(years)*	25–30	5
	31–35	13
	36–40	7
	41–45	8
	46–50	3
	Total	36
Educational qualification	No formal education	6
	Basic	8
	Secondary	12
	Tertiary	10
	Total	36
Sector of employment	Public	10
	Private	12
	Self-employed	14
	Total	36
Income *(GHS & $)*	Below 500 (Below $67)	2
	500–1000 ($67–$133.3)	5
	1001–1500 ($133.4–$200)	9
	1501–2000 ($200.1–$266.7)	14
	Above 2000 (Above $266.7)	6
	Total	36

NB! The exchange rate was based on ₵7.4–$1 of 19 May 2022

### Experiences of male’s support

Inquiry into the experiences of male involvement in antenatal-related care by married men revealed somehow low participation. Out of the 14 husbands interviewed, 9 of them had never accompanied their wives to antennal care while 6 of the pregnant women with delivery experience and the nursing mothers in addition to 4 of the pregnant women without delivery experience also disclosed that their husbands never accompanied them to antenatal care. These statements from the participants buttress the low male involvement in antenatal care.

*“What can I do for her if I accompany her to the hospital? I will just waste my time, so I never accompany her. I am required to provide money and other necessary things for her. If she needs help with antenatal care, my mother or her mother is always available. No need for me to support aside financial commitment.” (Participant 7, husband, 41 years).*

*“You know, when I got pregnant, my husband never supported me, just like when I conceived my earlier children. He goes to work early and comes home very late and even on weekends he is not around. All he knows is giving me money and expecting me to do things all by myself.” (Participant 19, pregnant woman with delivery experience, 33 years).*

*“I am with my first pregnancy and it is having a toll on me. My husband does not accompany me to antenatal care. This I do on myself, despite the desire to have my husband accompany to antenatal care.” (Participant 33, pregnant woman without delivery experience, 33 years).*

These responses pushed the researchers to probe from the husbands who do not support their wives, their willingness to support in antenatal care. All 9, but 4 husbands indicated the desire and willingness to support their wives during antenatal care in the future. The pregnant women with delivery experience and nursing mothers who never received any antenatal support from their husbands indicated their desire to receive such support but revealed their inability to discuss such issues with their husbands.

Furthermore, 5 of the husbands disclosed that they supported or currently support their wives with antenatal-related care, while this finding was supported by one pregnant woman without delivery experience, another pregnant woman with delivery experience and a nursing mother. Aside from antenatal care, other forms of male support during pregnancy were discovered including assisting with domestic chores, offering monetary support to the partner and regularly checking on their partners’ health. These statements have been extracted from the discourse with participants to that effect.

*“I am the firstborn of my parents and I saw how my father supported my mother during her pregnant periods. This I grew up with, so I always accompany my wife to antenatal and postnatal care. It is a basic duty I can perform for her and the unborn child” (Participant 2, husband, 36 years).*

*“Seeing my wife get tired with a heavy stomach makes me worried and compels me to accompany her to the hospital. Besides, whenever she’s pregnant I take over the running of the home by cooking, washing and doing other related chores. I enjoy accompanying her to antenatal and helping with house chores.” (Participant 12, husband, 45 years).*

As identified in the quotes, the husbands’ motive for supporting their wives was out of empathy owing to the stress of pregnancy on their wives. To some of the participants [husbands, pregnant women with delivery experience and nursing mothers alike], the support is premised on the perception of marriage as a partnership and children bearing as the responsibility of both males and females [husbands and wives].

### Facilitators of male participation

As clearly stated in the introduction, one of the objectives of the study is to examine the facilitators of husbands’ participation in antenatal-related care. Through the content analysis of the transcribed data, three major factors have been advanced as enablers of male’s participation in antenatal-related care. The first is the need to protect one’s family, the second is the changing gender roles and the third factor identified is the preferential treatment provided to pregnant women who attend antenatal clinics with their husbands. The subsequent paragraphs copiously discuss the aforementioned factors.

#### The need to protect one's family

A major facilitator of the husband’s participation in antenatal-related care as discovered in the study is the desire to protect their families. The husband or the father as ascertained in the study is required and obliged to protect his family [wife and children] since they are considered vulnerable. Almost all of the participants agreed that women are vulnerable during pregnancy and childbirth. The husband’s duty of protecting his wife and children is fully demonstrated in cases where the man perceives the woman to be prone to sickness, stress and fatigue. Therefore, the views of the husbands demonstrate that pregnancy is a period within which a man should offer protection and security for his partner. This implies the prioritization of the woman’s health by accordingly relieving her of performing certain difficult tasks such as cooking and washing for long hours among other household chores. Some of the men indicated that they cannot bear the pain their partners will go through if allowed to perform such difficult tasks. According to one of the husbands:

*“She (his wife) is hardworking and I know that. But during pregnancy, she is not in the best of shapes. Leaving her to go to antenatal all by herself is not the best. My concern for her health has made me accompany her always. I can only remember two occasions that I was unable to accompany her, although I asked my younger sister to accompany her.” (Participant 14, husband, 37 years).*

The importance of protecting one's family was also highlighted by two midwives. Specifically, the midwives stated instances where husbands explained leaving their work to attend to their wives at the hospitals since they want to protect them from any potential harm and stressful conditions.

*“To some men, they are willing and ready to protect their families. They leave their work and run up and down the whole place helping their wives. They so do it with joy, knowing they are protecting their wives. This help includes supporting them in their quest to receive antenatal care.” (Participant 28, male midwife).*

*“I once asked a married man who always accompanies his wife to the hospital the reason behind his action. He told me his wife had a miscarriage once due to stress and anxiety. Since then, he has always ensured he protects her from similar situations. This includes helping with household chores and bringing his wife to antenatal and postnatal clinic.” (Participant 25, female midwife).*

The findings demonstrate that taking one’s wife to antenatal care exhibits the compassion husbands have for their wives on one hand and the distribution of encumbrance associated with reproduction and protection of their families on the other hand.

#### Changing gender roles

Changing gender roles was also identified as a facilitator of the husband’s involvement in the care of their wives during pregnancy. Traditionally, men have been seen as leaders and providers of their family while women are expected to stay home, perform domestic chores and see to the care and wellbeing of the children and the family in their entirety. Evidence from the study, however, suggests that these socially constructed gender roles have altered over the past decades and in some cases, the differences have been blared. Notable in this changing gender role discussion is the economic contribution of women hitherto was seen as a man’s responsibility. This has compelled husbands and fathers to go beyond the provision of financial resources for the overall economic sustainability of the family to assist in domestic tasks beforehand viewed as the sole responsibility of the woman. These quotes from the participants provide more insights into the discussion.

*“Previously you know women were seen as baby-making machines and the men were providers of the family. Today the story is way different. We sometimes earn almost equal incomes, have higher education than them. So, it means they have to do more to show their care and support. We’re all equal despite our biological difference, so they understand and are willing to support us.” (Participant 20, nursing mother, 29 years).*

*“Things have changed in these few years. This is not what we have experienced at the time of our parents. Now there are these animals called gender equality and equity. Everyone is talking about it I think it is good. The effect is that you cannot treat your wife anyway you prefer. Even when she does not complain, people around you will point it out to you. So, we have to support them in everything as they support us, since there is nothing like this is a woman’s responsibility. Not even childbirth. We’re partners and they are not our slaves. Accompanying them to antenatal care is not beyond the scope of a husband’s responsibility” (Participant 8, husband, 44 years).*

Notwithstanding the evolution of gender roles during pregnancy and childbirth, the notion of gender-specific roles in the household persists. This is supported by the conceptualization of fathers as the principal breadwinners of their families while mothers are expected to perform house chores and reproductive roles, such as childbirth and child care duties. Amid the changing gender roles, some roles are still perceived to lean towards one of the genders than the other. Thus, the changing gender roles only imply a modification of some of the roles and not a total overhaul of the existing social conceptualization of gender roles.

#### Preferential treatment for wives who visit with husbands

The last but not least facilitator of males’ involvement in antenatal-related care identified in the Bosomtwe District of Ghana is the preferential treatment given to pregnant women who visit antenatal care with their husbands. It was discovered from both husbands, pregnant women with delivery experience, nursing mothers and midwives that preferential treatment or service is sometimes provided to couples who attend antenatal care. These couples are quickly attended to in most instances. Some of the midwives noted instances in which they asked pregnant women in a queue awaiting treatment to permit couples to be attended to in an attempt to encourage male involvement in antenatal care. Some of the pregnant women and the nursing mothers likewise alluded to being beneficiaries of such preferential treatment. These statements provide a more appropriate description of the scenario.

*“My husband and I always come for antenatal care because they attend to us quickly. You see I’m still here because he has not accompanied me today. Had he been here, I will have been attended to urgently since he has to go back to work.” (Participant 20, nursing mother, 29 years).*

*“I enjoy coming with my husband. The way you are treated in the presence of your husband is different from when you come alone. The health professionals become extra smart and vigilant and most importantly, they treat you with much care. You are spoken to humanely as against coming alone. You see, that is why I am here with my husband (pointing towards the direction of her husband in the process)." (Participant 22, nursing mother, 33 years).*

*“As an expectant mother with my first pregnancy, I love the support I am receiving from my husband, his involvement in antenatal care especially. He is so much involved such that he accords much attention to the instructions and recommendations given at hospital, by ensuring that I comply with all.” (Participant 32, a pregnant woman without delivery experience, 29 years).*

Thus, as a means of promoting the culture of male involvement in antenatal care in the District, some form of special treatment is given to couples who visit health centres. This, however, the participants reported that it is not widespread and thus requires much more effort to harness its potential. Aside from the preferential treatment two of the males who often accompany their wives to antenatal care indicated that much information and knowledge is acquired through pregnancy school organized for pregnant women. Explicitly, these ideas obtained from the pregnancy school has helped them ensure the health and safety of their wives and foetus before birth and the health of the child after birth.

### Barriers to male participation

Despite the low involvement of males in antenatal care, some facilitating factors influencing their engagement have been enlisted. Having presented evidence on the facilitators to the husband’s involvement in antenatal-related care, we now shift our attention to the barriers that hinder the process of males’ participation in antenatal-related care. In all, three broad themes were identified through the content analytical framework. Economic factors, cultural factors and health system factors were the identified barriers to the husband’s support during pregnancy. A detailed discussion of the results under each inhibiting factor is presented in the succeeding paragraphs.

#### Economic barriers

Husband’s competing job demands were reported by 7 of the husbands and 4 of the pregnant women with birth experience and nursing mothers as barriers to male’s involvement in antenatal-related care. Most of the husbands worked in formal and informal sectors [self-employed, public and private enterprises] in which it is difficult to make time and support their wives during pregnancy periods. This time constraint manifests in the husbands leaving home early and returning late, sometimes due to heavy traffic and the distance to work. They are unavailable to provide supportive services to their wives. Therefore, they are unable to take their wives to antenatal or help with household chores.

*“My husband works in a bank. He leaves very early and comes back late. He is unable to make time and take me to antenatal care. My other children are also young and I have to take care of them and attend antenatal care all by myself. Worse of it all is that he does not support the idea of having a maid to lessen the burden on me.” (Participant 16, nursing mother, 37 years).*

*“I have three children as of now and my wife is 6 months pregnant. Because of work, I spend less time with her. I cannot sacrifice work for antenatal care, after all, she has been doing that for the first three children so she can do it this time around.” (Participant 7, husband, 41 years).*

*“As you see now, I’m 8 months pregnant-pointing to her huge belly. My husband is in Accra and I am here. I haven’t seen him for weeks although we talk a lot. Coming to the hospital today, I had to struggle. The road is not good and I have to walk about 10–20 minutes before getting a vehicle. Life is stressful for us pregnant women without our husbands living close to us.” (Participant 19, pregnant woman with delivery experience, 33 years).*

The changing gender role as enlisted earlier as a facilitator of males' involvement in antenatal care of their wives has made women somehow autonomous in marriages and within the society. Nonetheless, men remained the expected providers of the family’s economic and or financial resources although women supplement their husbands’ contributions. The higher financial obligation on the husband thus hinders their efforts towards supporting their wives during pregnancy as a result of the attendant time constraint.

#### Cultural barriers

The identified cultural factors that hinder the effort of husbands towards supporting their wives during pregnancy and antenatal care are mockery from society and men’s perception of pregnancy and childbirth as a woman’s sole duty. In Ghana, males have been socialized into a system that projects masculinity in the light of leadership while feminine denote roles and responsibilities around reproduction and the performance of domestic chores. These cultural practices thus serve as barriers to the husband's support for their wives during pregnancy since childbearing is seen as a solitary duty of the woman. These quotes from the participants buttress these points.

*“My husband’s family thinks I want to overstretch him. They often argue that pregnancy and antenatal care is for women and men are supposed to work and support the wellbeing of the family. This pressure from his family has prevented him from accompanying me to antenatal care.” (Participant 36, a pregnant woman without delivery experience, 28 years).*

*“Day in day out, people see me as a fool. Simply because I always support my wife. They call me names and mock me whenever they see me. Some people think my wife controls me because I help and support her when she is pregnant, including escorting her to antenatal clinic. It discourages me sometimes, but I know what I’m doing so I will never give it up. I know one man who learnt that from me, but he could only implement it for about two months. He could not cope with societal pressure.” (Participant 6, husband, 36 years).*

*“Very often you see pregnant women in their third trimester with very young children at antenatal. When asked about their husband, you learn that due to societal pressure and negative orientation, they neglect their basic duty of supporting and protecting their family. For me, I think it is high time we relook at this whole idea of gender roles. The child is for both the father and the mother. Why should the mother be responsible for pregnancy care and postnatal care?” (Participant 29, Traditional birth assistant).*

It was further established through the interviews that couples who stayed in their building (built or rented flats) are more likely to have husbands supporting the wife during pregnancy than couples who live in compound houses. Again, the study found that couples who live with extended families are less likely to have husbands supporting the wife during pregnancy than couples who live outside their family house.

#### Health system barriers

Lack of services targeting men [husbands] at healthcare centres and hospitals was also a perceived barrier to male’s participation in antenatal care. The findings demonstrated that when husbands accompany their wives to antenatal care, they only sit and wait for long periods without involvement in any process. The absence of antenatal services targeted at husbands was also coupled with the understaffing of the health facilities which delays healthcare delivery. As earlier reported, time constraint hinders the involvement of males in antenatal care and as a result, longer waiting periods further demotivates men from taking part in antenatal care, especially in the absence of services targeted at men. These were reported below:

*“We are understaffed in this health centre. We have to run around doing a lot of things and attending to various cases. The waiting time for patients has increased and most husbands are unable to spend that much time at the hospital. Last time there was this pregnant woman who used to come to the health centre with her husband. But now he has stopped because he does nothing when he comes but wastes a lot of time waiting for his wife.” (Participant 27, female midwife).*

*“The way the antenatal care process is structured, little room is made for engaging the husbands. Most of the time we sit outside and wait for our wives. There were times I went to the consulting room but the health professionals never engaged me. When I tried asking questions, she was rude to me on the basis that she had a lot of patients to attend to while I was bothering her with such questions.” (Participant 3, husband, 33 years).*

*My husband must drive me to the hospital aside that nothing else. He sits in the car and waits for me. Sometimes he’ll call and tell me to alert him when they are done attending to me since he cannot wait there idle. I wished he could accompany me to the consulting room but he never does. Anyway, I don’t blame him, the place is full of women and as a man, and he might have felt a bit uneasy. A lot must be done to encourage our husbands to follow us, then that issue will be resolved.” (Participant 21, nursing mother, 34 years).*

It was established that the limited emphasis placed on the involvement of males in antenatal-related care amidst poor attitudes of healthcare providers and limited services directed at males at antennal clinics inhibits the participation of males in antenatal care. Some of the pregnant women with delivery experience and some nursing mothers indicated that healthcare providers do not encourage them to bring their husbands to the hospital or clinic while some of the husbands also reported similar viewpoints. These factors invariably possess challenges to the participation of males in antenatal-related care [especially antenatal care] among married men in the Bosomtwe District of Ghana.

## Discussion

The dearth of literature on the facilitators and barriers to the involvement of husbands in antenatal-related care in Ghana and for that matter Bosomtwe District in the Ashanti Region necessitated this qualitative probe into the subject matter. Probing interviews were conducted for husbands, pregnant women with and without delivery experience, nursing mothers, male and female midwives and traditional birth attendants (TBA) in the Bosomtwe District. Evidence showed lower participation of husbands in antenatal-related care among married men in the study area. Low participation of males in antenatal care has been reported by these scholars [32, 40, 41, 53] in their research into the subject matter. Vermeulen et al. [54] found low male involvement in antenatal care in Magu District, rural Tanzania. Although men perceived antenatal care as important for pregnant women, cultural barriers and gender roles served as barriers to their participation. This is however contrary to the finding of a study by Kabanga et al. [26] where they found that 56.9% of the pregnant women went to antenatal care with their male partners on their volition, and 51% were joined by male partners to antenatal care because the women had requested that their partners accompany them. Male partner attendance at antenatal care was substantially related to male partner awareness of antenatal care visiting dates [26]. We found that some of the wives were unable to discuss issues relating to antenatal care with their husbands. Contrary to our finding, in Tanzania, Kabanga et al. [26] noted that asking their husbands to accompany them to antenatal care led to a 51% antenatal care attendance rate among husbands. Within this framework, wives must endeavour to discuss, ask and encourage their husbands in the process. Though this would not be an easy undertaking, persistence on their part could be key in helping them reach that goal of involving their husbands in pregnancy care. 

Due the enormous benefits of male engagement in prenatal care on the health and well-being of pregnant women and their foetus [11, 37, 52], the low involvement rate in the Bosomtwe district of Ghana requires attention. This is particularly so in light of the 2030 agenda of reducing the global maternal mortality ratio to a rate lower than 70 per 100,000 live births,in addition to ending preventable deaths of new-borns and children under 5 years of age, reducing neonatal mortality to a rate lower than 12 per 1000 live births and under-5 mortality to a rate lower than 25 per 1000 live births. The attainment of these targets in Ghana requires pulling together the little factors that positively contribute to success as far these targets are concerned, while weakening the influence of the limiting factors. To that end, male’s involvement in antenatal care must be accorded significant attention, especially with the prevailing low experiences reported in the study.

The need to protect one’s family and changing gender roles were identified as facilitators of males’ involvement in antenatal care from the participants. This viewpoint has been espoused by previous studies [11, 41, 52]. These changing gender roles have replaced the traditional roles of women (reproducing and performing household chores) and the roles of men [leadership and financial support] [1, 11]. Nonetheless, while the performance of household chores is individually satisfying, doing so is at the expense of societal approval [14, 33, 36, 37]. Consequently, women and men tend to comply with dominant gender roles to gain societal acceptance. Results also indicate preferential treatment was provided to women who visited some health facilities with their husbands as a means of facilitating or encouraging male’s involvement in antenatal care. The motivation derived from the special treatment received by couples visiting antenatal care was also reported in earlier studies [38, 43].

In their study into strategies used to promote male involvement in antenatal care in Southern Tanzania, Peneza & Maluka [44] found health providers denying services to women attending antenatal care without their partners, and fast-tracking service to men attending antenatal care with their partners were some of the strategies. While our study confirmed this finding, it is rather retrogressive in the long run. The retrogressive nature of either fast-tracking antenatal services for pregnant women with their partners or denying access to antennal services for pregnant women without the partners, though may result in an initial increase in male’s involvement in antenatal care, will later support the patriarchal systems that in many ways limits and disempower women. To that end, the “evil in good” of the policy will exceed the intended purpose, thus posing serious threats to women empowerment. At best, it will result in “retrogressing” the positive and significant milestones achieved in lessening the potency of patriarchal systems and their deleterious impacts on women empowerment. Despite the significance of male engagement in pregnancy and childbirth-related services, this study finds that the usage and marketing of the male involvement policy should not accidentally hinder pregnant women's access to prenatal care services. Furthermore, programmes aimed at enlisting men's participation should be executed in ways that respect, encourage, and support women's choices and autonomy while also ensuring their safety [44].

Despite the changing gender roles, some men still perceive childbirth and its attendant responsibilities as solely the duty of the woman. This hindered their efforts to participate in antenatal care and in assisting with other ancillary responsibilities. This finding resonates with earlier studies that demonstrate cultural barriers serve or function as determinants of low male participation in antenatal care [26, 42, 54]. Continuous education and sensitization for lessening the potency of cultural factors and barriers to males support and involment in pregnancy care and antenatal care must be promoted in the district. Again, high job demands which hinder the involvement of men in antenatal-related care was also discovered and was in tandem with earlier studies [3, 27, 30]. Within the framework of the low male involvement, some health systems factors were identified as determinants. Health system factors such as the absence of services directed at husbands at health centres, long waiting times and attitude of health workers have been established in previous studies [16, 34, 49] as barriers to male participation in antenatal care. In this regard, health facilities must develop targeted interventions for husbands, to increase their involvement, engagement and participation.

### Strengths and weaknesses

The research was based on data obtained from many participants, including health practitioners, male partners [husbands], pregnant women [with or without delivery experience], and traditional birth assistants, which was a crucial strength of our study. This was an excellent chance to triangulate the data across the different participants. Despite this robustness, the study was conducted in only one district, and the findings may not adequately reflect the realities of other districts in Ghana. Consequently, we recommend large scale quantitative studies that investigate the phenomenon across multiple districts in Ghana for generalization. Furthermore, the study only identified the factors that facilitate or hinder male’s involvement in antenatal care. Being a qualitative study, we were unable to identify the relative importance of each factor. A quantitative study that regresses the influence of each band of facilitator and barrier to male’s involvement in antenatal care will not only address this gap, but offer prospects into initial the relative influence of each factor and the ones that must be accorded the greatest priority in policy and practice.

## Conclusion

Despite not being the direct receivers of antenatal care, the understanding of husbands is crucial in influencing women’s access to antenatal services. The importance of this knowledge and support is underscored by the husband's obligation and role as decision-makers [in most homes] with regards to where, how and when their partners seek health services. The elimination of barriers to male’s involvement in antenatal care is thus imminent and imperative. The provision of maternal health services that exclusively focus on women is defective bearing in mind the patriarchal society of the Bosomtwe District and by extension Ghana. The implementation of alternative strategies, for instance, couple counselling and prolonging operating times of health centres to cater for or to accommodate working men is recommended to provide a more accommodative and attractive avenue for men to support their wives during pregnancy. The District health officers and social workers must strengthen gender relations, enhance men’s appreciation of the family and social roles with regards to reproductive health while public education campaigns must be embarked on to engender males’ interest in providing supportive services to their wives aside financial obligations. These above-stated efforts must be reinforced by society in its entirety through disabusing the minds of people with regard to the perceived role of women as sole the bearers of pregnancy and childcare responsibilities in contrast to the perceived role of men as mere providers of financial resources.

## Data Availability

Data available from the corresponding author upon reasonable request.

## Abbreviations

MMDAs: Metropolitan, Municipal and District Assemblies
LI: Legislative instrument
CHPS: Community Health Planning Service
TBA: Traditional birth attendants
GHS: Ghana Cedis
SDGs: Sustainable Development Goals
